# Interaction with LC8 Is Required for Pak1 Nuclear Import and Is Indispensable for Zebrafish Development

**DOI:** 10.1371/journal.pone.0006025

**Published:** 2009-06-26

**Authors:** Christine M. Lightcap, Gabor Kari, Luis E. Arias-Romero, Jonathan Chernoff, Ulrich Rodeck, John C. Williams

**Affiliations:** 1 Department of Biochemistry and Molecular Biology, Thomas Jefferson University, Philadelphia, Pennsylvania, United States of America; 2 Department of Radiation Oncology, Thomas Jefferson University, Philadelphia, Pennsylvania, United States of America; 3 Cancer Genetics and Signaling Program, Fox Chase Cancer Center, Philadelphia, Pennsylvania, United States of America; 4 Department of Dermatology, Thomas Jefferson University, Philadelphia, Pennsylvania, United States of America; 5 Department of Molecular Medicine, Beckman Research Institute, City of Hope, Duarte, California, United States of America; Northwestern University, United States of America

## Abstract

Pak1 (p21 activated kinase 1) is a serine/threonine kinase implicated in regulation of cell motility and survival and in malignant transformation of mammary epithelial cells. In addition, the dynein light chain, LC8, has been described to cooperate with Pak1 in malignant transformation of breast cancer cells. Pak1 itself may aid breast cancer development by phosphorylating nuclear proteins, including estrogen receptor alpha. Recently, we showed that the LC8 binding site on Pak1 is adjacent to the nuclear localization sequence (NLS) required for Pak1 nuclear import. Here, we demonstrate that the LC8-Pak1 interaction is necessary for epidermal growth factor (EGF)-induced nuclear import of Pak1 in MCF-7 cells, and that this event is contingent upon LC8-mediated Pak1 dimerization. In contrast, Pak2, which lacks an LC8 binding site but contains a nuclear localization sequence identical to that in Pak1, remains cytoplasmic upon EGF stimulation of MCF-7 cells. Furthermore, we show that severe developmental defects in zebrafish embryos caused by morpholino injections targeting Pak are partially rescued by co-injection of wild-type human Pak1, but not by co-injection of mutant Pak1 mRNA disrupting either the LC8 binding or the NLS site. Collectively, these results suggest that LC8 facilitates nuclear import of Pak1 and that this function is indispensable during vertebrate development.

## Introduction

P21 activated kinase 1 (Pak1) is a serine-threonine kinase with important roles in cytoskeletal dynamics and cell motility. Increased Pak1 activity has been observed in advanced stages of breast, brain, pancreatic, ovarian, and colon cancers [1]. Forced expression of constitutively active Pak1 leads to increased proliferation and anchorage-independent growth of MCF-7 cells, a breast cancer cell line, whereas expression of a kinase dead Pak1 protein reduces the invasiveness of MDA-MB-435 breast cancer cells [2]. Furthermore, in transgenic mouse models, expression of activated Pak1 in breast epithelia is oncogenic, consistent with a functional role of Pak1 in tumor progression [3].

Pak1 is activated by Cdc42 and Rac1, members of the small GTPase family, and, in turn phosphorylates a wide range of targets with diverse functions. For example, phosphorylation of the estrogen receptor alpha by Pak1 at residue S305 increases its transactivation potential in a ligand-independent manner [4]. Pak1 also phosphorylates T261 of ErbB3 binding protein 1 (Ebp1), a transcriptional co-repressor that inhibits the growth of breast cancer cells. Specifically, upon phosphorylation, the repressor activity of Ebp1 is abolished, leading to increased proliferation of breast cancer cell lines [5]. Although much attention has been focused on roles of aberrant Pak1 activity in cancer, it has also become clear that Pak1 has critical roles in normal cell physiology and development, including mast cell function and the development of the central nervous system [6]–[8]. It is, however, currently poorly understood how different Pak1 phosphorylation events affect cell fate decisions in different tissues and cell types. In addition, while it is clear that Pak1 phosphorylates a large number of cytoplasmic and nuclear targets, it is unclear how Pak1 shuttles between cytoplasmic and nuclear locations.

In previous work, we [9] and others [10] have shown that Pak1 interacts with the dynein light chain, LC8, a small homodimeric protein best known for its participation in the assembly of the dynein motor complex. The LC8-Pak1 interaction has attracted significant interest as both Pak1 and LC8 appear to be coordinately upregulated in breast cancer specimens [10]. It has been proposed that Pak1 phosphorylates LC8 at serine 88 and that this event prevents BimL-dependent apoptosis in breast cancer by affecting LC8-BimL dimers [10]. However, recent studies demonstrated that the LC8-Pak1 interaction does not lead to LC8 phosphorylation, but rather represents a self-contained tetrameric complex that forms independently of the dynein motor protein [9]. Thus, the mechanism by which the LC8-Pak1 interaction affects either normal physiology or tumor development remains uncertain.

Here, we addressed whether LC8 serves a role in nuclear import of Pak1. This was based on the following observations and considerations. Pak1 enters the nucleus of MCF-7 cells after EGF stimulation, and among three potential nuclear localization sequences (NLSs) present in Pak1, only one appears to be critical for Pak1 nuclear import [11]. This site spans Pak1 residues 243–245, and thus is in close proximity to the LC8 binding sequence, which extends from residues 212–222 of Pak1 [9]. LC8 binding has previously been shown to facilitate nuclear import of other proteins, including the Rabies P protein and 53BP1 [12]. Although the molecular mechanism behind this function has remained obscure, blocking the LC8 interaction abrogates nuclear import of these targets [12]. We describe that (i) both the LC8 binding site and the NLS are required for nuclear import of Pak1 in breast epithelial cells, (ii) nuclear import of Pak1 by LC8 requires dimerization of the Pak1 NLS sequence and is independent of LC8's role in dynein transport, (iii) LC8-facilitated nuclear import is specific to Pak1 and is not seen with other Group 1 Pak kinases, and (iv) LC8-mediated dimerization and nuclear import functions of Pak1 are critically required for normal vertebrate, i.e., zebrafish, development.

## Results

### LC8 Facilitates Pak1 Nuclear Import in MCF-7 cells

**Figure 1:**
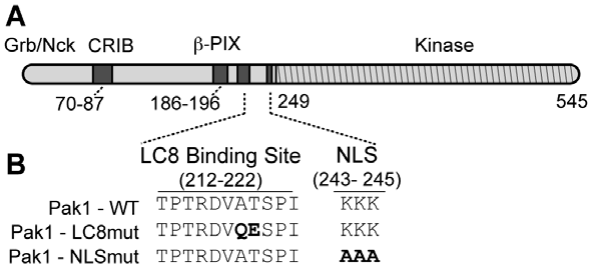
Functional domains of Pak1 constructs relevant to LC8 interaction and nuclear import. (A) Schematic representation of the domain structure of Pak1. The LC8 binding site and the nuclear localization sequence (NLS) shown to be critical for Pak1 nuclear import are highlighted. (B) Mutants used for immunofluorescent experiments with MCF-7 cells and reconstitution experiments in zebrafish. Mutations of A218Q and T219E in the LC8 binding site were generated to inhibit the LC8-Pak1 interaction and were based on the crystal structure 3DVP. The three lysine residues in the NLS were mutated to alanines.

Previous studies have established that Pak1 translocates into the nucleus upon EGF stimulation of MCF-7 breast cancer cells and that EGF-induced nuclear import requires a weak nuclear localization sequence consisting of residues 243 to 245 [11]. Further, LC8 has been shown to enhance nuclear import of both the Rabies P protein and the p53 binding protein 53BP1 [12]. Finally, our recent structural and biochemical studies showed that residues 212 to 222 of Pak1 encode an LC8 binding site adjacent to the nuclear localization site (Fig. 1A) [9].

**Figure 2:**
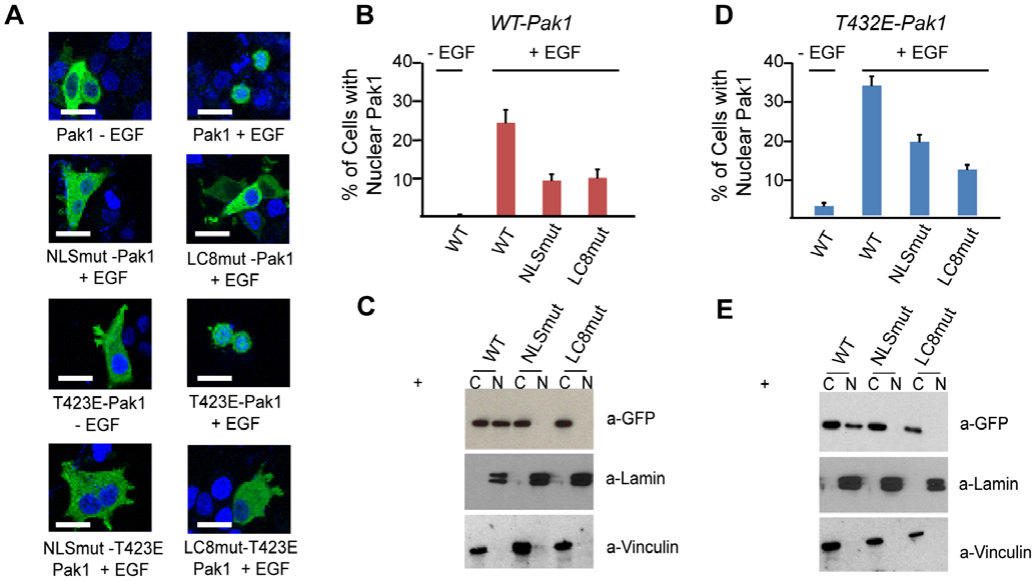
LC8 facilitates Pak1 nuclear import. (A) MCF-7 cells transiently transfected with either wild-type (WT), kinase active (T423E) GFP-Pak1, Pak1-LC8mut or Pak1-NLSmut mRNA. Scalebar shown is 10 microns. Mutations of either the NLS or the LC8 binding sequence in WT-Pak1 or T423E-Pak1 markedly reduced EGF-dependent nuclear import and stained with DAPI to visualize nuclei. (B) Quantification of nuclear accumulation of MCF-7 cells harboring either Pak1 or Pak1 mutants. Each bar represents percentage of cells with nuclear localized GFP (50 cells per experiment, done in triplicate). (C) The fraction of GFP located in cytoplasmic (C) and nuclear (N) fractions of MCF-7 cells after stimulation with EGF. Potential cross contamination of nuclear and cytoplasmic fractions was assessed by immunoblot analysis of Laminin A&C and Vinculin, respectively. (D) Nuclear import of T423E-Pak1 mutants after EGF stimulation. Nuclear percentages were calculated as in B. (E) Western Blot analysis of cytoplasmic and nuclear fractions of MCF-7 cells expressing T423E-Pak1 mutants after stimulation with EGF using an anti-GFP antibody.

Collectively, these results led us to test whether nuclear import of Pak1 depends on LC8 binding. We generated a double mutant Pak1 (A218Q and T219E [LC8mut-Pak1]; see Fig. 1B) to abrogate LC8 binding and tested whether these mutations would affect Pak1 nuclear import. Wild-type (WT) and LC8mut-Pak1 constructs were tagged N-terminally with GFP or Myc sequences. MCF-7 cells were transiently transfected with either GFP-WT-Pak1 or GFP-LC8mut-Pak1 and stimulated with EGF for 25 minutes. We observed markedly lower levels of nuclear GFP-LC8mut-Pak1 as compared to GFP-WT-Pak1 (Fig. 2). Co-immunoprecipitation experiments using MCF-7 cells that coexpressed the Myc-Pak1 mutant and HA-LC8 verified that LC8mut-Pak1 did not interact with LC8 whereas Myc-WT-Pak1 did (Fig. S1A). To confirm the role of the NLS in EGF-dependent nuclear import of Pak1, we mutated all three lysine residues in the Pak1 NLS sequence to alanines and evaluated nuclear Pak1 accumulation upon EGF stimulation (Figs. 1B and 2). Similar to the LC8mut-Pak1 construct, the NLS mutant, NLSmut-Pak1, also showed much less EGF-stimulated nuclear accumulation (Fig. 2). Quantitative analysis of Pak1 nuclear accumulation by confocal microscopy revealed that 24% of the EGF-stimulated cells transfected with GFP-WT-Pak1 contained GFP in the nucleus, in contrast to only 8% or 10% of EGF-stimulated MCF-7 cells transfected with the GFP-LC8mut or GFP-NLSmut Pak1 constructs, respectively. Unstimulated MCF-7 cells transiently transfected with any of the three constructs contained GFP in less than 1% of nuclei.

To confirm these results independently we prepared nuclear and cytoplasmic fractions of the EGF-stimulated, transfected MCF-7 cells and used immunoblot analysis to assess the GFP content in these fractions. As expected, we did not detect GFP in the nuclear extracts of cells expressing the LC8mut-Pak1 and NLSmut-Pak1 constructs but observed comparable levels of nuclear and cytoplasmic GFP in the WT-Pak1 expressing cells. Western blot for GFP indicated that all GFP-Pak1 constructs were expressed at comparable levels (Fig. S1B).

In previous work, we showed that LC8 preferentially binds to dimeric targets [13]. Pak1 appears to be homodimeric when inactive, with the inhibitory switch domain binding to the C-terminal lobe of the Pak1 kinase domain, and positioning the kinase inhibitory segment in the cleft of the kinase active site [14]. Upon binding of Cdc42 or Rac1 to the CRIB domain, this inhibition is released, allowing Pak1 to autophosphorylate and presumably become an activated, monomeric species [15]. In a recent study, NMR and hydrodynamic studies showed that the kinase domain of Pak2 (93% identity to the Pak1 kinase domain) forms a homodimer; however, phosphorylation of the activation loop renders a monomeric species [16]. Thus, it is not clear whether the inactive or activated form of Pak1 binds LC8. To begin to address this issue, we generated the kinase active form, T423E, and tested whether LC8 was important for Pak1 nuclear localization of this Pak1 variant. We observed that, similar to WT-Pak1, the constitutively active form of Pak1 was cytosolic, but translocated to the nucleus as soon as 20 min after EGF stimulation (Figs. 2A and 2D). Co-immunoprecipitation experiments revealed that the T423E/LC8mut Pak1 construct also does not interact with LC8 (Fig. S1A). Mutation of the nuclear localization sequence similarly reduced the nuclear accumulation of constitutively active Pak1. Western blot analysis of cytoplasmic and nuclear fractions from cells transiently transfected with T423E-Pak1 constructs further established the requirement of LC8 for Pak1 nuclear import (Fig. 2E).

### LC8 Facilitates Pak1 Nuclear Import Independent of its Association with Dynein

LC8 is frequently assumed to bridge cargo to the dynein motor complex for retrograde transport, and the requisite binding of LC8 for nuclear import is consistent with dynein-mediated, retrograde transport. However, our recent structural and thermodynamic data suggest that LC8 regulates the function of its target proteins in a dynein-independent manner [13]. Specifically, homodimeric LC8 preferentially binds dimeric targets—either dynein or another of its dimeric targets. Moreover, we recently showed that Pak1 binds to the same groove on the LC8 surface as the dynein intermediate chain and that Pak1 binding to monomeric LC8 is very weak (100 to 200 µM) [9]. Thus, to achieve an appreciable concentration of the complex under physiological conditions, we propose that a dimeric form of Pak1 must bind to a dimeric form of LC8. We further propose that LC8 binds to and/or stabilizes a conformation of Pak1 that is necessary for nuclear import and that this event is independent of the function of LC8 in the dynein complex.

**Figure 3:**
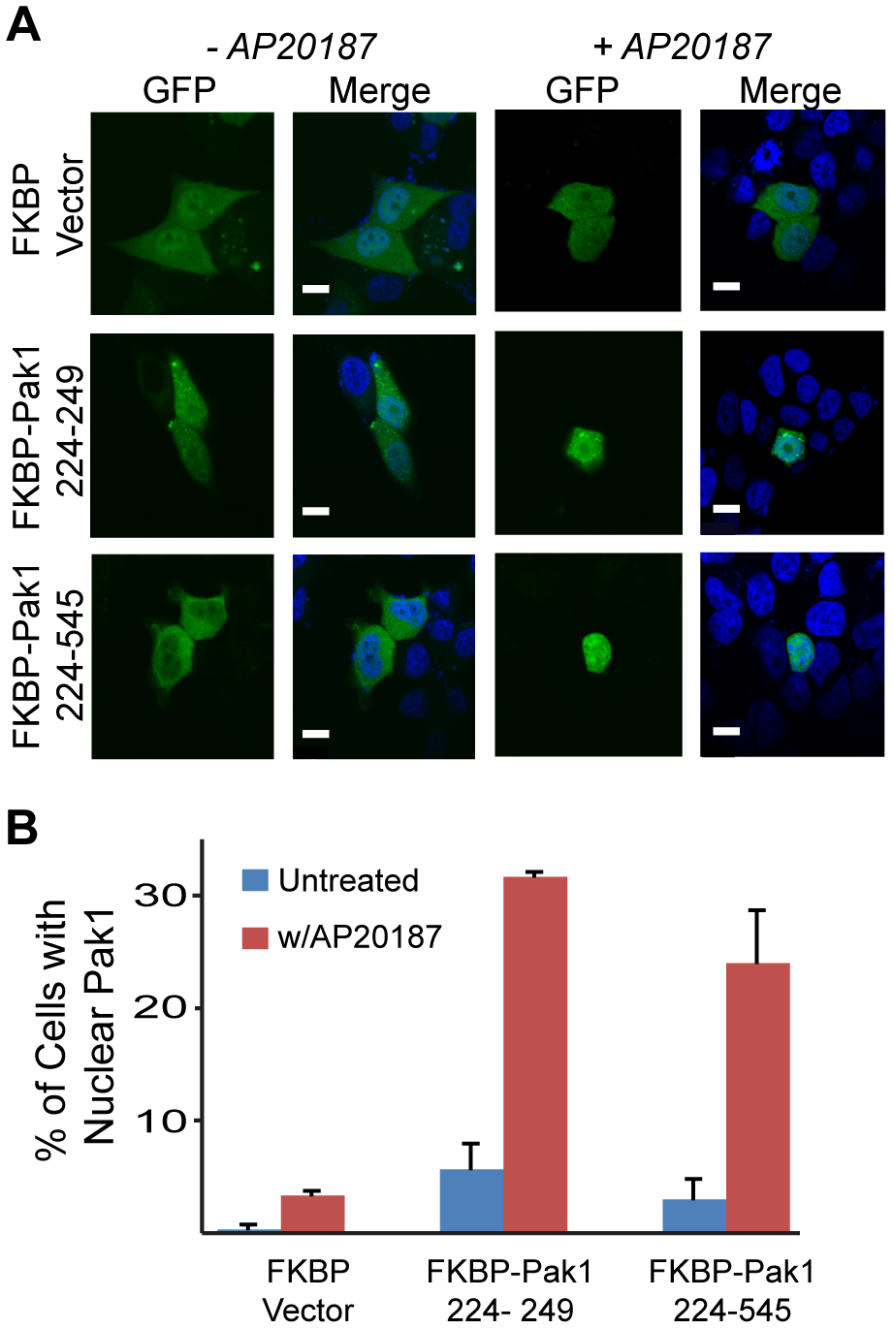
Pak1 nuclear import is contingent upon LC8 dimerization and is dynein independent. (A) Confocal microscopy images of MCF-7 cells transiently transfected with either the FKBP vector, Pak1 226–249, or Pak1 226–545 show either GFP-Pak1 construct is cytoplasmic in the absence of AP20187 and stained with DAPI to visualize nuclei. Scalebar shown is 10 microns. (B) Quantification of confocal images indicates an ∼5 fold increase in nuclear GFP over non-treated cells upon AP20187 treatment. Data quantified in the same manner as in Fig. 2B.

To test this hypothesis and provide independent evidence that LC8 induces a conformational change in Pak1, we turned to the FKBP/AP20187 inducible dimerization system to mimic LC8 binding to Pak1 [17]. Specifically, we fused FKBP to the N-terminal region of Pak1 that immediately follows the LC8 binding region that encodes the nuclear localization sequenc http://www.plosone.org/article/info3Adoi2F10.13712Fjournal.pone.0004640e (residues 226–249) and to a similar construct that includes this region as well as the kinase domain (residues 226–545). In addition, we fused GFP to the N-terminus of FKBP to facilitate examination of the subcellular localization of the expressed constructs. All three constructs, GFP-FKBP-Pak1(226–249), GFP-FKBP-Pak1(226–545), and a GFP-FKBP control were transfected independently into MCF-7 cells and each construct was located in the cytoplasm before the addition of a chemical dimerizer, AP20187 (Fig. 3A). Upon the addition of AP20187 and in the absence of EGF, a substantial fraction of both GFP-FKBP-Pak1 products translocated to the nucleus within 25 min. The level of nuclear localization for the shorter GFP-FKBP-Pak1(226–249) construct was similar to that of the constitutively active, GFP-T423E-Pak1 (32% vs. 34%, respectively). In addition, the nuclear accumulation of the longer construct, GFP-FKBP-Pak1(226–545), after induction by AP20187 was comparable to that of GFP-WT-Pak1 stimulated with EGF (24% in both experiments). These results are consistent with the view that a specific Pak1 dimeric conformation enabled by an LC8 dimer or LC8 surrogate (i.e., FKBP) regulates Pak1 nuclear import in a dynein-independent fashion.

### LC8 interaction is unique to Pak1 in the Pak family

The Pak family of kinases consists of six members divided into two groups, Group 1 and Group 2 Paks [18]. This classification is based on structural and sequence similarities and functional differences between the six kinases. Pak1, Pak2 and Pak3 encompass the Group 1 Pak kinases, and all three members contain an auto-inhibitory domain, which is released upon binding of Cdc42/Rac1 to the CRIB domain, and a β-PIX binding site, an interaction previously shown to be important for Pak activation [19]. Each member also encodes an NLS sequence immediately preceding the kinase domain; however, Pak1 and Pak2 contain identical NLS sequences consisting of three sequential lysine residues (Fig. S2A). The C-terminal region of Pak2 (213–524) can translocate into the nucleus after cleavage by Caspase 3 in response to apoptotic stimuli [20]. To date, Pak3 has only been identified in the cytoplasm. The Group 2 Paks differ from Group 1 Paks in that the NLS signal is located at the far N-terminus, and they lack a β-PIX binding site [21]. We did not observe a definitive LC8 binding site in Group 2 Paks.

Because Pak2 can also translocate to the nucleus, we generated a GFP-Pak2 construct and tested whether EGF stimulation of transfected MCF-7 cells led to its nuclear accumulation. GFP-Pak2 was entirely cytoplasmic, and remained cytoplasmic when treated with EGF as in the GFP-Pak1 experiments (Fig. S2B). Of note, apoptotic events that activate Caspase 3 lead to the cleavage of Pak2 between residues 212 and 213, and only the C-terminal Pak2 fragment that contains the NLS and the kinase then translocates to the nucleus [20]. Although MCF-7 cells are Caspase 3-deficient and the GFP protein was fused to the N-terminus of Pak2, we immunoblotted for GFP before and after stimulation with EGF and showed that Pak2 is not cleaved in these experiments (Fig. S2C).

Collectively, these results indicate that LC8 specifically interacts with and enhances nuclear import of Pak1, but not Pak2, in EGF-stimulated cells. Furthermore, Pak3, although highly homologous to the other Group 1 Pak members, does not contain a clear LC8 binding site and we predict that its nuclear localization is not LC8 dependent.

### Pak1 nuclear import is critical for survival and development

Elevated expression levels of Pak1 and LC8 in tumor tissues and growth of MCF-7 cells overexpressing Pak1 and LC8 in soft agar assays suggest that the LC8-Pak1 interaction is critical for tumor progression. Pak1 has also been shown to phosphorylate nuclear targets, including estrogen receptor alpha, PFK-M and SHARP, consistent with a role of nuclear Pak1 in disease progression [1], [11]. However, it is not clear whether the LC8-Pak1 interaction is relevant or necessary under physiological conditions. To address this question, we turned to zebrafish as a model organism. The Pak1 orthologue in zebrafish has 81% sequence identity and 87% sequence conservation with the human Pak1 protein. In addition, the LC8 binding site in the zebrafish Pak1 protein has 73% sequence identity and 91% sequence conservation with the human Pak1 protein (Fig. S3A). Of particular importance is an aspartate at the identical position in the LC8 binding region in both the zebrafish and human Pak1 proteins. Our previous studies indicate this specific aspartate interacts with an absolutely conserved hydrogen bond network in LC8 and is critical for the stability of the LC8-Pak1 interaction [9]. Finally, the zebrafish LC8 sequence is 93% identical and 98% similar to human LC8.

**Figure 4:**
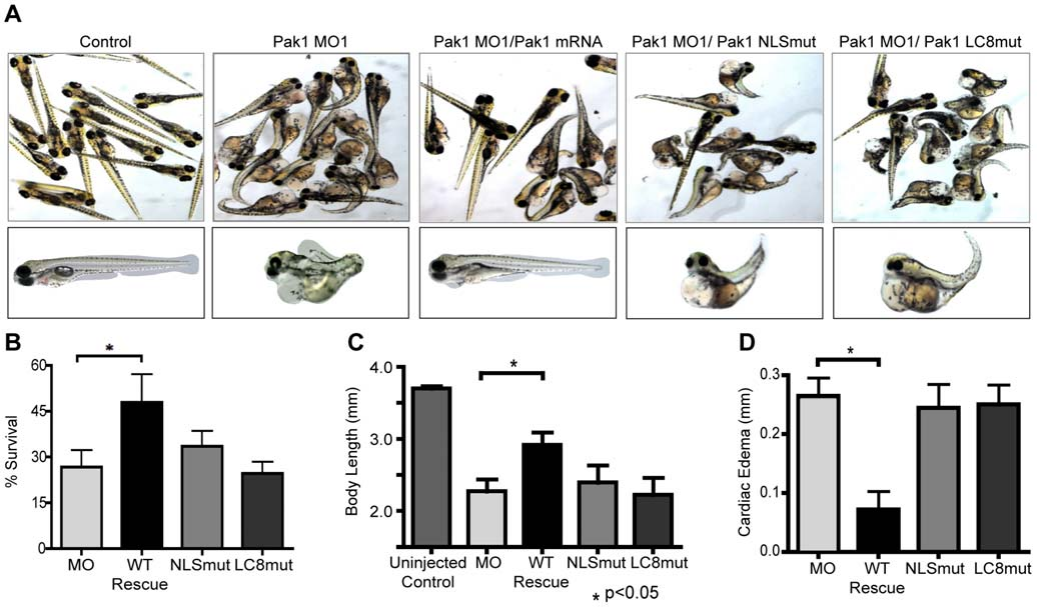
Pak1 nuclear import is critical for zebrafish development and survival. (A) Representative images of zebrafish embryos at 4 dpf. Embyros were co-injected with the morpholino (MO1) and either human wt-Pak1 mRNA, human Pak1-NLSmut or human Pak1-LC8mut (last three panels). Uninjected embryos and MO1 only injected embryos are also shown. (B–D) Quantitative analysis of differential zebrafish survival, body length, and extent of cardiac edema at 4 dpf in embryos injected with different morpholino/Pak1 mRNA combinations. For B, percent survival of uninjected control fish is approximately 90%. For D, cardiac edema for experimental fish was normalized to the control fish.

To determine the effect of Pak1 knockdown, we designed a Pak1-specific morpholino that targets Pak1 mRNA sequences surrounding the ATG start codon. We first injected 60 fertilized embryos with the Pak1 morpholino (MO1) (concentrations ranging from 0.125 to 2 mM) and observed a decreased rate of survival and morphological alterations in surviving embryos associated with increasing concentrations of MO1 compared to the normal mock-injected embryos. Differences were seen as early as 24 hours post-fertilization (hpf) and included smaller heads and eyes, shorter body lengths, and, by 48 hpf, pericardial edema. At 96 hpf, these malformations progressed to include curled tails, marked pericardial edema, and gross morphological defects in the heart (Fig. 4A). By measuring the length of the body axis, we observed statistically significant (p<0.05) reductions in overall body length in Pak1 MO-injected fish when compared to uninjected embryos. Using the transgenic line Tg:VEGFR2-GRCFP [22], we further observed impaired, delayed angiogenesis in embryos injected with MO1. Specifically, lumen formation of intersegmental vessels was markedly reduced, leading to visibly reduced blood flow in the trunk and tail (data not shown). It remains to be investigated whether these abnormalities reflect a functional role of Pak1 in angiogenesis or occur secondary to impaired heart development.

Several control experiments were performed to ascertain whether the effects observed were specific to Pak1 knockdown. First, we designed a second morpholino that targets a different sequence in zebrafish Pak1 mRNA, a 5′ intron/exon splice site (MO2). As expected, injecting embryos with MO2 (0.5–1 mM) markedly reduced viability and led to aberrations in body axis development and pericardial edema in a fashion similar to the ATG-targeted Pak1 MO1 (Fig. S3B). In addition, we attempted to rescue developmental defects caused by Pak1-targeted MOs by co-injecting Pak1 mRNA. In these experiments, we used human Pak1 mRNA because there is significant sequence identity and similarity between the zebrafish and human orthologs. Embryos injected with both Pak1 MO1 and hPak1 mRNA showed a 53% survival rate compared to the 28% survival rate of MO1-only injected fish (Fig. 4B). Significantly, 80% of the surviving co-injected fish were morphologically indistinguishable from the control fish. On the other hand, all surviving MO1-only injected embryos showed extensive pericardial edema and other malformations by 4 dpf (Fig. 4D).

To distinguish putative roles of Pak1 nuclear import *in vivo*, we generated mRNA of the nuclear import deficient mutants and co-injected embryos with mutant mRNAs and Pak1 MO1 at the same concentration as the WT-Pak1 recovery experiments. Embryos were injected with Pak1 MO1 and either NLSmut-Pak1 or LC8mut-Pak1 mRNA. Neither mRNA was able to rescue the phenotype associated with Pak1 MO1 injection (Fig. 4A). Embryos injected with NLSmut-Pak1 mRNA had a 28% survival rate at 4 dpf, similar to that of the group injected with MO1 alone (Fig. 4B). Furthermore, embryos injected with LC8mut-Pak1 mRNA had an 18% survival rate, further indicating that the LC8-Pak1 interaction is necessary during development.

**Figure 5:**
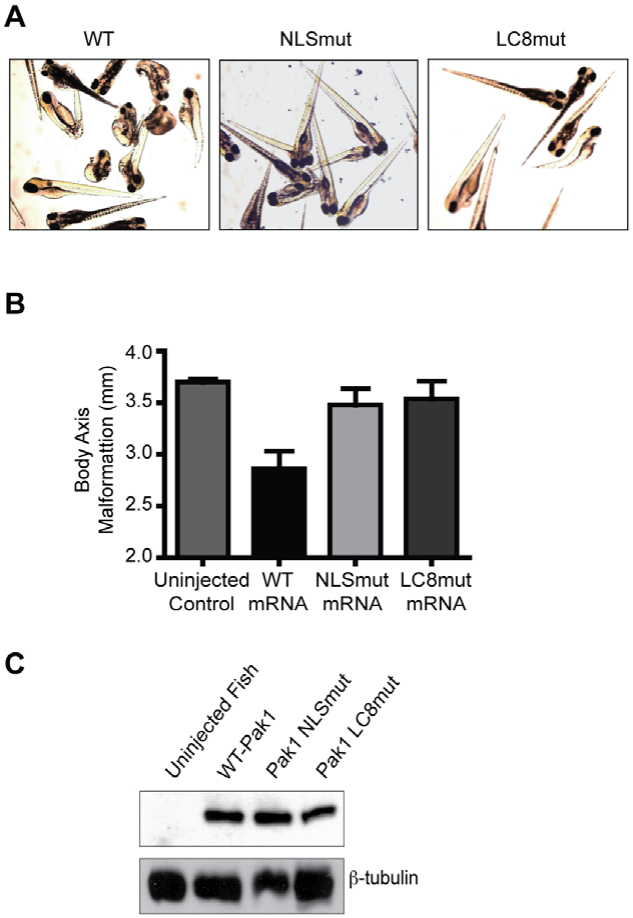
Overexpression of human Pak1 produces embryonic abnormalities in zebrafish that are contingent upon nuclear import of Pak1. (A) Images of zebrafish embryos at 4 dpf. Embryos were injected with either Pak1 WT-mRNA or one of the mRNA of the Pak1 nuclear import mutants (80 ng/µL). (B) Quantitative analysis of differential zebrafish body axis malformation at 4 dpf in embryos injected with either Pak1 WT-mRNA or one of the mRNA of the Pak1 nuclear import mutants. (C) Immunoblot analysis of lysates from zebrafish injected with either human Pak1-WT, NLSmut, or LC8mut mRNA.

Next, we examined the effects of overexpressing human Pak1 in zebrafish embryos. Injection of hPak1 mRNA alone led to death and/or severe morphological aberrations in the injected embryos. Interestingly, these were similar to the aberrations observed in fish treated with Pak1-targeted MOs, and mainly consisted of aberrations in body axis development and pericardial edema. However, embryos singly injected either with the human NLSmut- or LC8mut-Pak1 mRNA showed very few morphological alterations, consistent with the view that deleterious effects of Pak1 overexpression on zebrafish morphology and survival depend on nuclear import (Figs. 5A and B). Western blot analysis using a Pak1 specific antibody verified that all three groups of human Pak1 mRNA injected fish expressed similar levels of Pak1 protein (Fig. 5C). In aggregate, these results support a crucial role of nuclear import of Pak1, facilitated by LC8, in development and survival of zebrafish embryos.

## Discussion

The evidence presented here supports the following conclusions: (i) nuclear import of Pak1 requires interaction with LC8, (ii) this interaction occurs independently of the LC8/dynein interaction, (iii) among the Pak family members, this function is specific to Pak1, and (iv) LC8-mediated nuclear import of Pak1 is essential during development of zebrafish embryos.

We previously reported that LC8 and Pak1 form a dimer of dimers and that Pak1 binding precludes simultaneous interaction of LC8 with either the dynein complex or other cargo [9]. Previous studies by others have shown that Pak1 exists as a homodimer when inactive [14]. The results presented here extend this concept, and suggest that activated Pak1 similarly exists in a dimeric state enabled, at least in part, by interaction with LC8. This finding was surprising as it has been shown that Cdc42 binds to the inactive and presumably dimeric Pak1, permitting its autophosphorylation and producing an active monomeric state [15]. Similarly, recent NMR studies of the Pak2 kinase domain indicate that the non-phosphorylated kinase also forms a homodimer; however, phosphorylation of the activation loop produces a monomeric kinase [16]. Notwithstanding these earlier reports, our results are consistent with the view that at least a fraction of activated Pak1 is dimeric and that this dimeric state is stabilized, in part, by LC8 interaction.

These observations raised the question whether LC8-bound dimeric Pak1 has any identifiable functions in cell biology. Here, we provide evidence that dimerization of Pak1 enabled by sequences N-terminal to the NLS promotes the nuclear import of Pak1. Specifically, the short fragment of Pak1 containing the NLS sequence (residues 226–249), when fused to FKBP in place of the LC8 binding domain, remained cytosolic despite being unencumbered by steric restraints from the remaining domains of wild-type Pak1. However, upon dimerization by adding AP20187, this short fragment immediately dimerized and translocated to the nucleus of MCF-7 breast cancer cells. This is consistent with the hypothesis that the dimeric state of Pak1, recognized by LC8, is necessary to create a ‘bipartite’ NLS complex recognizable by an importin. This situation is not without precedent, as biochemical and structural studies have shown that nuclear localization of the Stat family members and SREBP-2 similarly requires dimerization [23], [24].

**Figure 6:**
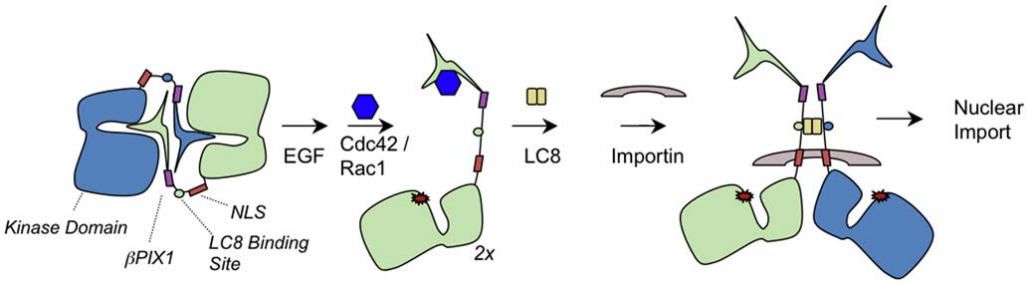
Model of Pak1 translocation. Pak1 is a stable homodimer in its inactive state. Multiple signals from different pathways (GPCRs, RTKs, and lipids) act on Pak1 and activate it. One potential pathway suggests that the N-terminus of Pak1 binds to Nck or Grb2, which is associated with the activated EGFR. This permits Cdc42/Rac1 to bind to Pak1, allowing it to trans-autophosphorylate. We propose this permits LC8 binding, which localizes the weak NLS to act as a bipartite ligand for importin binding and ultimately nuclear translocation.

Taken together, these observations suggest that EGF-induced nuclear localization of Pak1 requires a significant conformational change accompanying the transition from the inactive-to-active state, dimerization through LC8-binding sequences in close proximity to the NLS, and alignment of weak NLS signals in Pak1 monomers through LC8-dependent orientation followed by importin recognition and nuclear import (Fig. 6).

To better understand functional implications of Pak1 nuclear import, we turned to zebrafish embryos as a facile vertebrate model system. First, we established by use of Pak1-targeted antisense oligonucleotides that Pak1 expression is essential for normal development of zebrafish. Second, we demonstrated that human Pak1 mRNA partially rescued embryonal lethality and embryonal malformations caused by Pak1 knockdown. Third, we observed that mutating either the LC8 binding site or the NLS site abrogated the capacity of human Pak1 to rescue the defects caused by zebrafish Pak1 knockdown. While it is currently unclear which target cells and tissues are responsible for the phenotypes caused by Pak1 knockdown, these results provide strong, independent evidence that LC8-mediated Pak1 nuclear import has a central role in zebrafish development and potentially in mammalian cells and tissues. This role goes beyond the regulation of estrogen receptors, as previously reported [4], and suggests that additional nuclear Pak1 targets are involved in development.

In conclusion, this study provides the first evidence that Pak1, localized in the nucleus, has an essential role in development. It further reinforces the notion that the dimerization state of Pak1 among other signal transducers has functionally significant consequences for nuclear import and that LC8 plays an essential role in stabilizing Pak1 dimerization.

## Materials and Methods

### Materials

Anti β-Actin, anti-GFP, anti-Laminin A/C, anti-Myc, and anti-HA antibodies were purchased from Santa Cruz Biotechnology. Anti-Vinculin and anti-Pak1 antibodies were from Cell Signaling. EGF and the anti-β tubulin antibody were from Sigma. The Pak1 morpholino (MO) (GeneTools, LLC) was designed to target the ATG start site (5′-CCTCTACTTCCCCATTGTCTGACAT-3′). A second MO, MO2, was designed to target the Pak1 5′ intron/exon splice site (5′-GCATCACTCACTCTTGTCTCCTC-3′).

### Expression Plasmids and Transfection

Pak1 (Accession Number: NP 002567) was subcloned into pEGFP-C1 vector (Clontech) and pCS2 vector (Dave Turner, University of Michigan). The Pak1 mutants (K299R, T423E, LC8mut, and NLSmut) were generated using the Quikchange site directed mutagenesis kit (Stratagene). The FK506 binding protein (FKBP) gene and Pak1 226–249 or Pak1 226–545 were cloned into pEGFP-C1-FKBP vector. For transient expression, cells were transfected using Lipofectamine (Invitrogen).

### Immunofluorescence

MCF-7 human breast cancer cells on glass coverslides (Fisher) at 1×10^6^ cells per 6-well tissue culture plate were transfected with vectors containing different Pak1-GFP constructs. After 24 h, cells were serum starved for 14–16 h before being stimulated with EGF (100 ng) for 20 min. For the FKBP experiments, cells were treated with AP20187 (100 nM, Ariad) in serum-free media for 30 min. Cells were fixed with 4% paraformaldehyde and nuclei were counterstained with 4′,6-diamidino-2-phenylindole (DAPI; Sigma).

### Cell Fractionation, Western Blotting and Co-Immunoprecipitation

Cellular fractionation experiments were performed as previously described [25], MCF-7 cells in 10 mm plates were transfected with 5 µg Myc-Pak1, or Pak1 mutants, and 5 µg HA-human LC8 for co-immunoprecipitation experiments and. Cells were lysed, loaded on Protein A agarose beads, washed and separated on reducing SDS-PAGE gels. Gels were immunoblotted with the corresponding antibody.

### Zebrafish experiments

Maintenance of zebrafish stocks and embryo collection were carried out following standard procedures [26] and with approval by the Institutional Animal Care and Use Committee at Thomas Jefferson University. Morpholino experiments were performed as previous described (see supplemental data) [27], Fisher's Exact Test was performed using a sample sizes of n = 120 embryos per condition. The p value was set for less then 0.001 with a 95% confidence interval.

Additional details are provided in the supplemental (Methods S1).

## Supporting Information

Methods S1 Extended methods and supplemental figure captions
(0.08 MB PDF)

Figure S1 Binding assays and expression of Pak1 constructs (A) Co-immunoprecipitation of whole cell lysates overexpressing HA-LC8 and Myc-Pak1, and associated mutants. Results indicate mutation of the LC8 binding site in Pak1 abrogates the interaction. (B) Western blot of GFP-Pak1 constructs in MCF-7 cells.
(0.28 MB PDF)

Figure S2 Pak2 is not cleaved after EGF Stimulation. (A) Sequence alignment of Group1 Pak kinases, highlighting that the Pak1 LC8 binding site (boxed) is absent in Pak2 and Pak3. By contrast, Pak1 and Pak2 share identical nuclear localization sequences (NLS) positioned at the same location upstream of the kinase domain. (B) Representative examples of subcellular distribution of Pak2 as determined by confocal microscopy. Pak2 does not translocate to the nucleus after EGF stimulation in MCF7 cells. (C) Western blot of MCF7 cells expressing either GFP alone or GFP-Pak2 before and after stimulation with EGF. Results show GFP runs at the same molecular weight after EGF treatment, showing that Pak2 is not cleaved in these experiments.
(0.42 MB PDF)

Figure S3 Zebrafish Pak1 Protein and Rescue. (A) Sequence alignment of Human and Zebrafish Pak1 protein. (B) Pak1 knockdown with a Pak1 MO to the 5′ intron/exon splice site (MO2) showed phenotypes identical to the Pak1 MO for the initial ATG codon. Co-injection of human Pak1 mRNA was able to recover the phenotype. Pictures were taken at a 12.5× magnification. (C) Quantification of zebrafish survival at 4 dpf in embryos injected with Pak1 MO2 and embryos rescued with human Pak1 wt-mRNA.
(0.30 MB PDF)
