# Supplementary material for: Interaction with LC8 Is Required for Pak1 Nuclear Import and Is Indispensable for Zebrafish Development
Source: PLoS One. 2009 Jun 26;4(6):e6025. doi: 10.1371/journal.pone.0006025 (PMC2698211; doi:10.1371/journal.pone.0006025)
Supplement: Methods S1 — Extended methods and supplemental figure captions (0.08 MB PDF) [file pone.0006025.s001.pdf]

Methods.

Expression Plasmids and Transfection

Pak1 (Accession Number: NP 002567) was subcloned into the BglII and EcoRI sites in the pEGFP-C1 vector and the BamHI and EcoRI sites of the pCS2 vector. The FKBP protein was cloned into the Sac1 and EcoR1 sites of the pEGFP-C1 vector. Pak1 226–249 and Pak1 226–545 were then cloned into the SpeI and EcoRI sites of the pEGFP-C1-FKBP vector. These constructs were verified using automated sequencing by the Kimmel Cancer Center sequencing facility. Human LC8 (Accession Number: NP 001032584) was isolated as a yeast-two hybrid partner of Pak1, and subcloned as a BamHI/EcoRI fragment into pJ3H (1). Pak1 was cut with SalI and EcoRI and cloned into the XhoI and EcoRI sites of pMIG-w vector. Colonies were tested for Myc expression using western blot analysis.

Cell Maintenance and Immunofluorescence Experiments

MCF-7 human breast cancer cells were cultured in Dulbecco's modified Eagle medium (Gibco) supplemented with 10% fetal bovine serum (Invitrogen). Images were taken with a Nikon C1 Spectral Confocal microscope and LSM Image Browser software using a 60X objective lens and a Cascade 650 (Photometrics) monochrome camera.

Co-Immunoprecipitation

For the co-immunoprecipitation experiments, cells were lysed in RIPA buffer (20 mM Tris (pH 7.5), 150 mM NaCl, 1 mM EDTA, 1 mM EGTA, 1% Triton X-100, 2.5 mM sodium pyrophosphate, 1 mM β-glycerophosphate, 1 mM Na_3VO_4, 1 µg/mL Leupeptin, 1 mM PMSF). Equal amounts of protein were incubated with anti-HA or anti-Myc antibodies (1:50 ratio) overnight. Lysates were loaded on Protein A agarose beads, washed five times with RIPA buffer before being loaded and separated via SDS-PAGE

Zebrafish Survival and Morphological Analysis

For analysis of MO effects on zebrafish survival and gross morphology, dechorionated embryos (96 hpf) were anesthetized (1:100 dilution of 4 mg/ml tricaine methanesulfonate; Sigma) on a glass depression slide. Morphology was assessed visually using a light transmission stereo microscope (Leica Mikroscopie and Systeme GmbH Wetzlar, Germany) at 12.5X magnification, and representative images recorded using an MZFIII stereomicroscope or Axioplan2 microscope with an Axiocam camera and AxioVision software (version 3.0.6.1). Embryos were photographed in 1X-PBST. Similarly, survival of embryos was assessed visually at 24 h intervals up to 96 hpf by light microscopy. The criterion for embryonic survival was the presence of cardiac contractions.

1. Sells, M. A. & Chernoff, J. (1995) *Gene* **152,** 187-9.

Supplemental Figure 1. Binding assays and expression of Pak1 constructs (A) Co-immunoprecipitation of whole cell lysates overexpressing HA-LC8 and Myc-Pak1, and associated mutants. Results indicate mutation of the LC8 binding site in Pak1 abrogates the interaction. (B) Western blot of GFP-Pak1 constructs in MCF-7 cells.

Supplemental Figure 2. Pak2 is not cleaved after EGF Stimulation. (A) Sequence alignment of Group1 Pak kinases, highlighting that the Pak1 LC8 binding site (boxed) is absent in Pak2 and Pak3. By contrast, Pak1 and Pak2 share identical nuclear localization sequences (NLS) positioned at the same location upstream of the kinase domain. (B) Representative examples of subcellular distribution of Pak2 as determined by confocal microscopy. Pak2 does not translocate to the nucleus after EGF stimulation in MCF7 cells. (C) Western blot of MCF7 cells expressing either GFP alone or GFP-Pak2 before and after stimulation with EGF. Results show GFP runs at the same molecular weight after EGF treatment, showing that Pak2 is not cleaved in these experiments.

Supplemental Figure 3. Zebrafish Pak1 Protein and Rescue. (A) Sequence alignment of Human and Zebrafish Pak1 protein. (B) Pak1 knockdown with a Pak1 MO to the 5' intron/exon splice site (MO2) showed phenotypes identical to the Pak1 MO for the initial ATG codon. Co-injection of human Pak1 mRNA was able to recover the phenotype. Pictures were taken at a 12.5x magnification. (C) Quantification of zebrafish survival at 4 dpf in embryos injected with Pak1 MO2 and embryos rescued with human Pak1 wt-mRNA.
