# Supplementary figures and images for: Interaction with LC8 Is Required for Pak1 Nuclear Import and Is Indispensable for Zebrafish Development
Source: PLoS One. 2009 Jun 26;4(6):e6025. doi: 10.1371/journal.pone.0006025 (PMC2698211; doi:10.1371/journal.pone.0006025)

Supplemental Figure 1

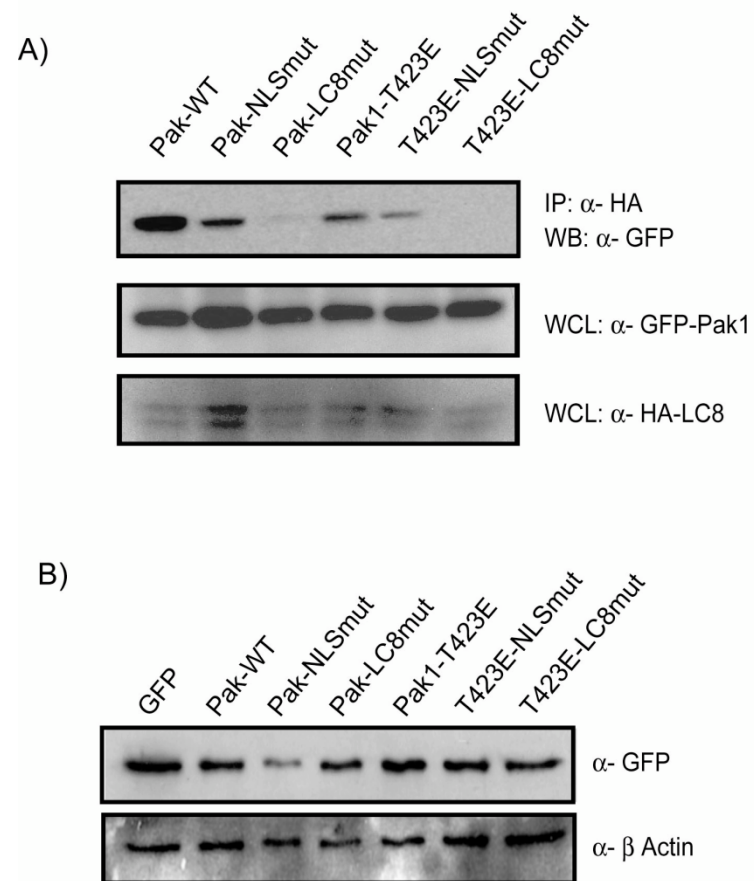

Supplement: Figure S1 — Binding assays and expression of Pak1 constructs (A) Co-immunoprecipitation of whole cell lysates overexpressing HA-LC8 and Myc-Pak1, and associated mutants. Results indicate mutation of the LC8 binding site in Pak1 abrogates the interaction. (B) Western blot of GFP-Pak1 constructs in MCF-7 cells. (0.28 MB PDF) [file pone.0006025.s002.pdf]

Supplemental Figure 2

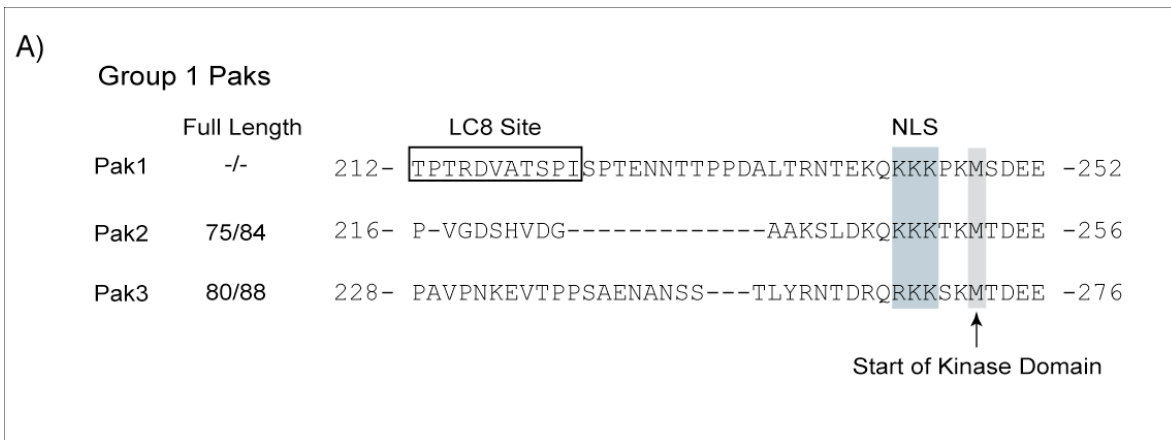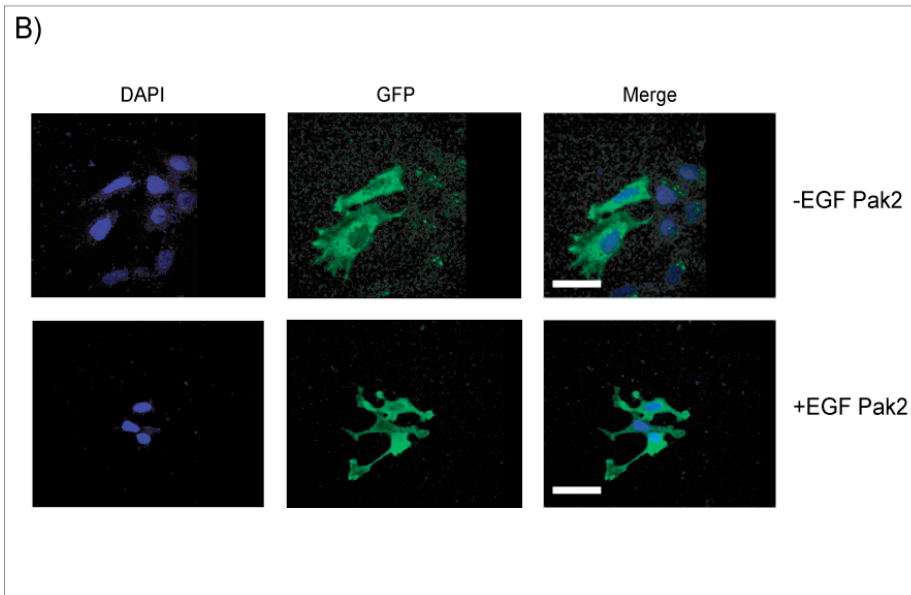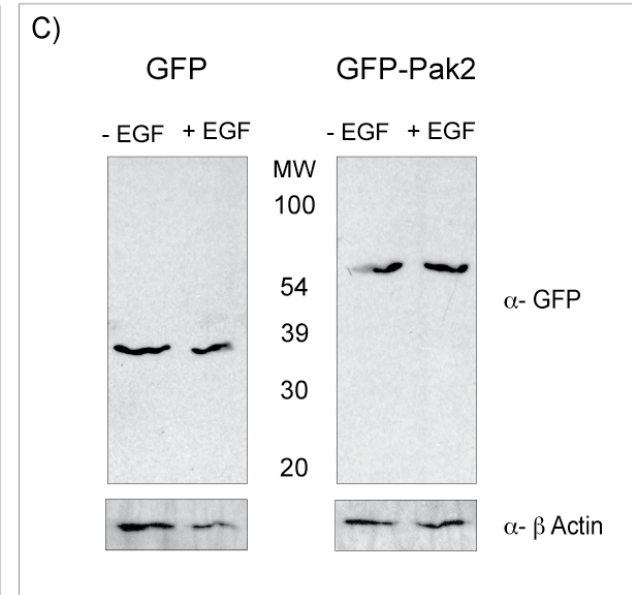

Supplement: Figure S2 — Pak2 is not cleaved after EGF Stimulation. (A) Sequence alignment of Group1 Pak kinases, highlighting that the Pak1 LC8 binding site (boxed) is absent in Pak2 and Pak3. By contrast, Pak1 and Pak2 share identical nuclear localization sequences (NLS) positioned at the same location upstream of the kinase domain. (B) Representative examples of subcellular distribution of Pak2 as determined by confocal microscopy. Pak2 does not translocate to the nucleus after EGF stimulation in MCF7 cells. (C) Western blot of MCF7 cells expressing either GFP alone or GFP-Pak2 before and after stimulation with EGF. Results show GFP runs at the same molecular weight after EGF treatment, showing that Pak2 is not cleaved in these experiments. (0.42 MB PDF) [file pone.0006025.s003.pdf]
