# Supplementary material for: Interaction with LC8 Is Required for Pak1 Nuclear Import and Is Indispensable for Zebrafish Development
Source: PLoS One. 2009 Jun 26;4(6):e6025. doi: 10.1371/journal.pone.0006025 (PMC2698211; doi:10.1371/journal.pone.0006025)

Supplemental Figure 3

A)

Pak1

	Full Length		LC8 Binding Site	
H. sapiens	(--/--)	212	TPTRDVATSPI	222
D. rerio	(81/87)	213	TSTKDAATSPI	222

B)

Uninjected Controls | Pak1 MO2 | Pak1 MO2/ human mRNA

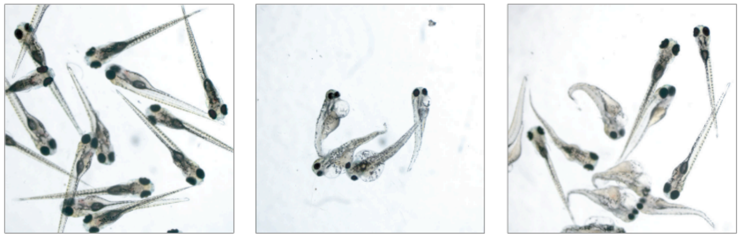

C)

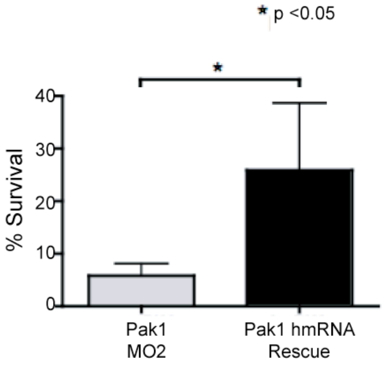

Supplement: Figure S3 — Zebrafish Pak1 Protein and Rescue. (A) Sequence alignment of Human and Zebrafish Pak1 protein. (B) Pak1 knockdown with a Pak1 MO to the 5′ intron/exon splice site (MO2) showed phenotypes identical to the Pak1 MO for the initial ATG codon. Co-injection of human Pak1 mRNA was able to recover the phenotype. Pictures were taken at a 12.5× magnification. (C) Quantification of zebrafish survival at 4 dpf in embryos injected with Pak1 MO2 and embryos rescued with human Pak1 wt-mRNA. (0.30 MB PDF) [file pone.0006025.s004.pdf]
